# Hyperenhancement of LA Wall by Three-Dimensional High-Resolution Late Gadolinium-Enhanced MRI and Recurrence of AF After Catheter Ablation

**DOI:** 10.3390/jcm13237357

**Published:** 2024-12-03

**Authors:** Minako Kagimoto, Shingo Kato, Ryouya Takizawa, Sho Kodama, Keisuke Suzurikawa, Mai Azuma, Naoki Nakayama, Kohei Iguchi, Kazuki Fukui, Masanori Ito, Tae Iwasawa, Tabito Kino, Daisuke Utsunomiya

**Affiliations:** 1 Department of Cardiology, Saiseikai Yokohamashi Nanbu Hospital, Yokohama 234-0054, Japan; minako@yokohama-cu.ac.jp; 2 Department of Diagnostic Radiology, Yokohama City University Graduate School of Medicine, Yokohama 236-0004, Japan; d_utsuno@yokohama-cu.ac.jp; 3 Department of Cardiology, Kanagawa Cardiovascular and Respiratory Center, Yokohama 236-0051, Japan; takizawa.1c10n@kanagawa-pho.jp (R.T.); sk.num10.y@gmail.com (S.K.); k.suzurikawa@gmail.com (K.S.); mai.smile.mai2@gmail.com (M.A.); nakan@yokohama-cu.ac.jp (N.N.); iguchi.0h30i@kanagawa-pho.jp (K.I.); fukui.0o400@kanagawa-pho.jp (K.F.); 4 Department of Diagnostic Radiology, Kanagawa Cardiovascular and Respiratory Center, Yokohama 236-0051, Japan; kcrc.rt.m.itoh@gmail.com (M.I.); tae_i_md@wb3.so-net.ne.jp (T.I.); 5 Department of Cardiology, Institute of Medicine, University of Tsukuba, Tsukuba 305-8575, Japan; kino-tabito@unim.ac.jp

**Keywords:** atrial fibrillation, late gadolinium enhancement, fibrosis, catheter ablation

## Abstract

**Background/Objectives**: This study investigated the relationship between LA (LA) enhancement on three-dimensional (3D) late gadolinium enhancement (LGE) MRI and recurrence after catheter ablation in patients with AF (AF). **Methods**: A total of one hundred patients with AF (mean age: 68 ± 9 years, 50% with paroxysmal AF) were included in this study. Each patient underwent a high-resolution 3D LGE MRI prior to catheter ablation, allowing for detailed imaging of the LA wall. Quantitative analysis of the enhancement was performed using dedicated software designed for volumetric measurements of LA LGE. Recurrence of AF was monitored over a 90-day period following the ablation procedure. The primary outcome was the correlation between the volume of LGE in the LA and the recurrence of AF. **Results**: Multivariate analysis confirmed that the volume of LA LGE, defined as the volume exceeding 1SD above the mean signal intensity of the LA, was an independent predictor of recurrence [hazard ratio: 1.16 (95%CI: 1.04–1.29, *p* = 0.0057)]. The area under the curve for recurrence prediction using 3D LGE MRI was 0.74 (95%CI: 0.63–0.86), with an optimal threshold of 11.72 mL, providing a sensitivity of 55% (95%CI: 32–77%) and a specificity of 86% (95%CI: 77–93%). **Conclusions**: LA enhancement assessed by high-resolution LGE MRI may serve as a valuable imaging marker for predicting the recurrence in patients with AF following catheter ablation.

## 1. Introduction

Catheter ablation for atrial fibrillation (AF) remains challenging, and the recurrence of atrial arrhythmias is common even after multiple procedures [1,2]. In particular, treating persistent AF is difficult, and despite various additional ablation techniques being developed alongside pulmonary vein isolation, the recurrence rate remains high [3,4,5]. Left atrial (LA) fibrosis is a characteristic of atrial damage seen in AF and plays an important role in the pathophysiology of AF [6,7]. Progression of LA fibrosis, as evaluated by late gadolinium-enhanced (LGE) magnetic resonance imaging (MRI), has been reported to be independently associated with the recurrence of atrial arrhythmias after ablation [8,9]. In other words, greater residual fibrosis is associated with worse post-procedural outcomes, emphasizing the role of fibrotic cardiomyopathy as a substrate for maintaining arrhythmias.

The volume of LA wall fibrosis, as assessed by three-dimensional LGE-MRI, has been shown to be associated with a higher rate of AF recurrence after catheter ablation [10]. A previous study evaluated left atrial hyperenhancement using MRI in 260 patients with atrial fibrillation [8]. The unadjusted hazard ratio for recurrent arrhythmia per 1% increase in left atrial fibrosis was 1.06 (95%CI, 1.03–1.08; *p*  <  0.001), indicating that the risk of recurrence increased as the stage of fibrosis progressed [8]. In these previous studies, the evaluation of high-signal regions in the LA was performed using quantitative analysis of three-dimensional (3D) LGE. However, the methodology and details of the software employed were not clearly defined, posing challenges for its application in clinical practice [8,9,10]. One previous study proposed a more convenient method for quantitatively evaluating high-signal regions in the LA using clinically available software [11]. This method involved assessing the severity of fibrosis through fusion images of 3D LGE and dynamic contrast-enhanced LA imaging. However, there are no data on the relationship between high-signal regions in the LA obtained through this method and the recurrence of AF.

In this study, we investigated the relationship between LA fibrosis, quantitatively evaluated using fusion images of 3D LGE and dynamic contrast-enhanced LA imaging, and the recurrence of AF after catheter ablation.

## 2. Materials and Methods

**Figure 1:**
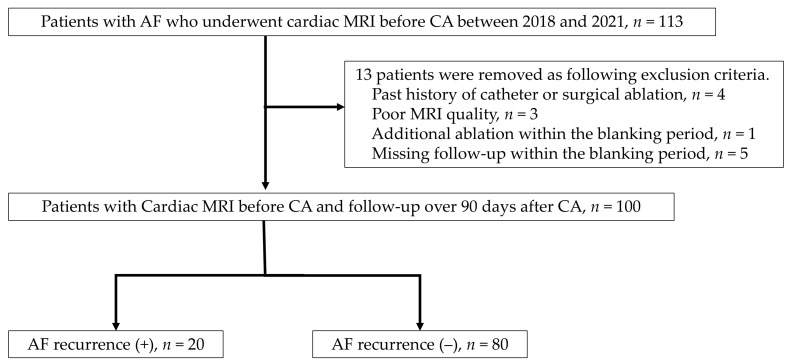
Flow chart of patients with atrial fibrillation for catheter ablation with cardiac MRI.

This study is a retrospective analysis of cardiac MRI data from a single institution. The inclusion criteria consisted of AF (AF) patients who underwent cardiac MRI before catheter ablation (CA). The exclusion criteria included cases with poor MRI quality (*n* = 3), cases with a history of catheter or surgical ablation (*n* = 4), cases that dropped out during the blanking period (90 days after CA) (*n* = 5), and cases that underwent CA for any type of tachycardia within the blanking period (*n* = 1) (Figure 1). This study was approved by the ethics committee of the Kanagawa Cardiovascular and Respiratory Center (approval number: KCRC-20-0054), and informed consent was obtained from all participants using the opt-out method.

### 2.1. Imaging Protocol for Contrast Enhanced Magnetic Resonance Angiography and 3D LGE MRI

The contrast-enhanced imaging was conducted using a 1.5-tesla MR scanner (Intera Achiva; Philips Medical Systems, Best, The Netherlands) equipped with a 32-channel cardiac coil. Gadobutrol (Bayer HealthCare Pharmaceuticals, Whippany, NJ, USA) was injected at a dosage of 0.1 mL (mmol)/kg during the first pass of contrast. Contrast-enhanced magnetic resonance angiography (MRA) was captured with an axial 3D turbo field echo (TFE) sequence, without ECG gating, and using exhalation breath-hold to assess the morphology of the pulmonary veins and LA (LA). Key imaging parameters included a repetition time (TR)/echo time (TE) of shortest/shortest, a voxel size of 1.37 × 1.37 × 3.00 mm^3^ (reconstructed to 0.68 × 0.68 × 1.50 mm^3^), a flip angle of 35°, and sensitivity encoding (SENSE) factors of 2 for the phase direction and 1.5 for the slice direction.

Following the initial scan, LGE-MRI was performed 10 min after contrast injection. This was done using a 3D inversion-recovery T1-TFE sequence, equipped with respiratory navigation and ECG gating in the transverse plane. Standard imaging parameters for DE-MRI included a TR/TE of shortest/shortest, a voxel size of 1.25 × 1.26 × 2.60 mm^3^ (reconstructed to 0.63 × 0.63 × 1.30 mm^3^), a flip angle of 15°, and an inversion time ranging from 280 to 330 ms. The T1 value of the left ventricular myocardium was determined using the Look–Locker method to identify the T1 null point. For patients in sinus rhythm, imaging was performed during the mid-diastolic phase of the left ventricle, while for those in AF, the shortest trigger delay for cardiac synchronization was applied. Fat suppression techniques were employed to eliminate fat signals. These imaging protocols were developed based on previously established methods [11].

### 2.2. Assessment of LA Hyperenhancement

**Figure 2:**
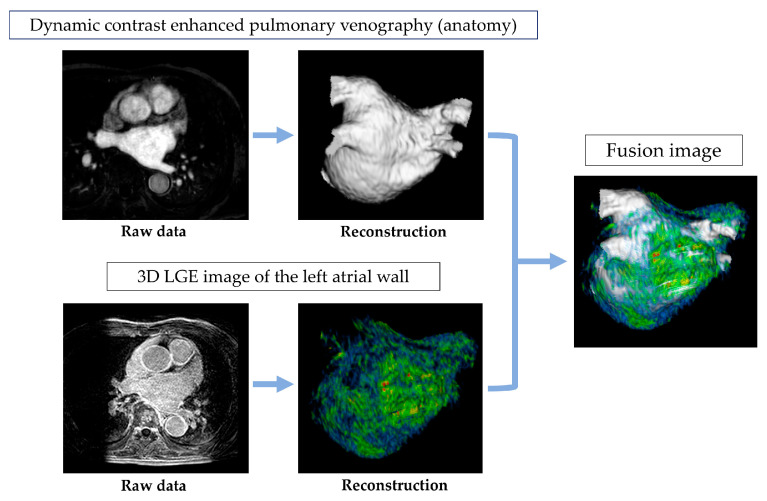
Acquisition and analysis of MRI images.

The source images from CE-MRA and DE-MRI were uploaded to a workstation (Ziostation 2; Ziosoft, Tokyo, Japan), where a 3D reconstruction of the CE-MRA was generated, following the approach used in previous studies. This image was then combined with a high-resolution LGE MRI to evaluate fibrosis in the LA. For fibrosis quantification, the contrast enhancement intensity was measured. A voxel intensity histogram analysis of the LA wall was conducted, setting the threshold for intensity at 1 standard deviation (SD) above the mean voxel intensity. Additionally, the intensities were categorized using a color-coded scale, with blue representing values below 1SD, green for 1–2SD, yellow for 2–3SD, and red for values exceeding 4SD [11] (Figure 2).

### 2.3. Ablation Procedure and Evaluation of AF Recurrence

All patients underwent pulmonary vein isolation, and additional ablation was performed at the discretion of the attending physician. After the ablation, the recurrence of AF was evaluated using Holter monitoring and 12-lead electrocardiograms. AF recurrence was defined as any episode of AF, atrial flutter, or atrial tachycardia lasting at least 30 s and occurring after the 90-day blanking period.

### 2.4. Statistical Analysis

Statistical analysis was performed using SPSS Statistics version 29 (IBM, Armonk, NY, USA) and R version 4.4.1 (The R Foundation for Statistical Computing, Vienna, Austria). For comparison between the recurrence and non-recurrence groups, a paired *t*-test was used for normally distributed data, and the Mann–Whitney U test was applied for non-normally distributed data. Cox proportional hazards univariate and multivariate analyses were performed to evaluate predictors of AF recurrence. Receiver Operating Characteristic (ROC) analysis was also conducted to assess the predictive ability of recurrence. The optimal cutoff value was calculated using the Youden index. Predictors of recurrence were calculated using Cox proportional hazards multivariate analysis. Statistical significance was defined as *p* < 0.05.

## 3. Results

### 3.1. Patients’ Characteristics

The patient characteristics are shown in Table 1. The mean age was 68 ± 9 years. Males accounted for 78% of the patients, and the average body mass index was 24.3 ± 3.6 kg/m^2^. Regarding the type of AF, half of the patients had paroxysmal AF, while the remaining 50% had either persistent or long-standing persistent AF. The mean CHADS2 score was 1.5 ± 1.1. The average LA volume index measured by echocardiography was 49.7 ± 20.9 mL/m^2^. When comparing the recurrence group and the non-recurrence group, there was a significantly higher proportion of patients with persistent or long-standing persistent AF in the recurrence group. Additionally, the duration since the onset of AF was longer, and the LA volume index was significantly larger in the recurrence group (all *p* value < 0.05).

**Figure 3:**
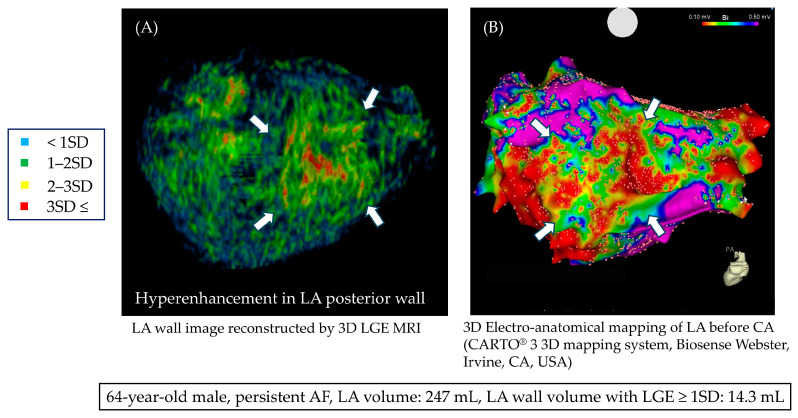
Three-dimensional LGE MRI in a case of persistent AF; (**A**) LGE MRI shows an area of enhancement on the posterior wall (white arrow). (**B**) In the 3D voltage map during catheter ablation, a low-voltage area was observed at the site corresponding to the enhancement seen in the LGE MRI.

Table 2 shows a summary of the cardiac MRI parameters. The mean LA volume was 137.0 ± 46.8 mL. When comparing the recurrence group with the non-recurrence group, both the volume of LGE of the LA exceeding 1SD (11.8 ± 4.4 mL vs. 8.6 ± 2.7 mL, *p* < 0.001) and 2SD (1.6 ± 1.1 mL vs. 1.0 ± 0.8 mL, *p* = 0.010) were statistically significantly higher in the recurrence group. Figure 3 shows representative images from a recurrence case. The patient is a 64-year-old male with persistent AF, and there is a massive area of LGE observed in the LA. The voltage map for this case is also shown, where a red low-voltage area can be seen on the posterior wall. Supplemental Table S2 shows a comparison of patient backgrounds between those with LA LGE ≥ 1SD and < 1SD.

### 3.2. Association Between LA LGE and AF Recurrence

**Figure 4:**
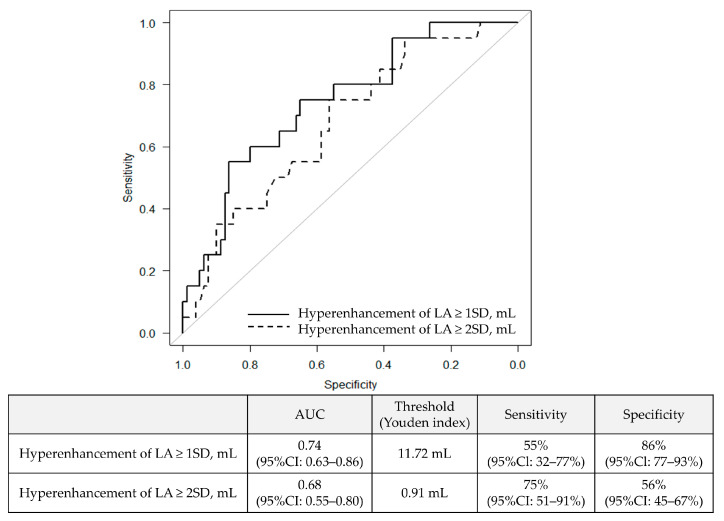
Receiver operating characteristic curve of LA wall volume with hyperenhancement for predicting recurrence.

**Figure 5:**
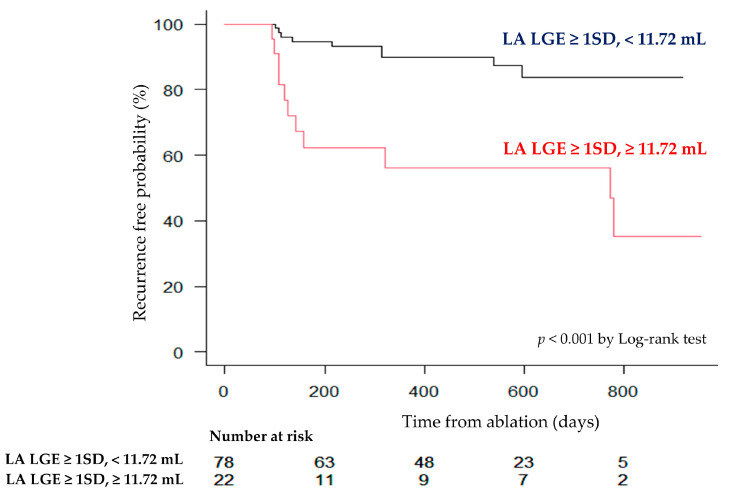
Kaplan–Meier curve for recurrence after CA stratified by an optimal threshold of LA wall hyperenhancement.

Details regarding the additional ablation methods are summarized in Supplemental Table S2. The mean follow-up period was 464 days. The longest follow-up period was 955 days. During that time, 20% of patients experienced recurrence of AF. Table 3 shows the results of the univariate Cox regression analysis regarding AF recurrence. The type of AF, time since onset, CHADS_2_ score, LA volume as assessed by echocardiography, LA volume measured by MRI, LA wall volume, and the volume of LGE exceeding 1SD and 2SD were found to be statistically significant factors (all *p*-value < 0.05). In the multivariate analysis, the type of AF (persistent or long-standing persistent) [3.54 (95%CI: 1.10–11.4, *p* = 0.033)] and the LGE volume exceeding 1SD [1.16 per mL (95%CI: 1.04–1.29, *p* = 0.0057)] were identified as significant predictors of AF recurrence (Table 4). Figure 4 shows the ROC analysis results for AF recurrence. The area under the curve (AUC) for the volume of LGE exceeding 1SD was 0.74 (95%CI: 0.63–0.86), and for the volume exceeding 2SD, the AUC was calculated as 0.68 (95%CI: 0.55–0.80). When calculating the Youden index, the optimal cutoff value for the volume exceeding 1SD was 11.72 mL, with a sensitivity of 55% (95%CI: 32–77%) and a specificity of 86% (95%CI: 77–93%). Figure 5 shows the Kaplan–Meier curve stratified by the cutoff value of 11.72 mL for the volume exceeding 1SD. Patients with a LA LGE volume greater than 11.72 mL had a statistically significantly higher recurrence rate compared to those with a smaller volume.

## 4. Discussion

The main results of this study are as follows: (1) Quantitative evaluation of LA LGE was feasible using clinically available software. (2) The volume of LA LGE exceeding 1SD was identified as an independent predictor of AF recurrence. These findings suggest that the quantitative evaluation of LA LGE may be useful in predicting AF recurrence after AF ablation.

The recurrence of AF remains a significant therapeutic challenge, and its prediction and prevention are fraught with difficulties [12,13,14]. Particularly, catheter ablation, being an invasive procedure with associated risks of complications, necessitates an accurate assessment of the recurrence risk. The pivotal role of LA fibrosis in the pathophysiology of AF has been demonstrated through postmortem tissue analyses and histological evaluations of surgical specimens obtained during open-heart procedures. Postmortem pathological studies have confirmed a correlation between LA fibrosis and the history of AF [15]. Moreover, fibrosis in atrial tissue, evaluated from specimens obtained during surgery, has been shown to predict postoperative recurrence [16]. Thus, there is a well-established body of evidence linking LA fibrosis with the severity of AF.

Cardiac MRI is a diagnostic modality that excels in evaluating myocardial tissue, offering a non-invasive assessment of myocardial infarction and fibrosis [17,18]. Recent studies employing high-resolution 3D LA LGE have assessed areas of abnormal enhancement, indicating the severity of fibrosis, and have demonstrated their association with AF recurrence [8,9,19]. Additionally, more severe left atrial LGE has been associated with an increased risk of major adverse cardiovascular and cerebrovascular events (MACCE), with the increased risk primarily driven by a higher incidence of stroke or transient ischemic attack (TIA) [10]. Furthermore, left atrial fibrosis demonstrates a weak correlation with global strain, and its relationship with functional indicators has also been recognized [19]. These studies provided groundbreaking findings, underscoring the potential of MRI in this context. However, the software used in these studies for assessing LA fibrosis was in-house, and the details of its analytical mechanisms and workflow were unclear, posing challenges for generalizing the results. Subsequently, studies using clinically available software for the evaluation of LA fibrosis and the visualization of ablation areas have been published [11,20]. In our study, we focused on the potential of this method, conducting evaluations in a larger patient cohort, and demonstrated the relationship between LA fibrosis and postoperative recurrence of AF. In this study, we found that the volume of high-intensity regions in high-resolution LA LGE exceeding 1SD was associated with an increased risk of AF recurrence. Another appealing aspect of this method is its ability to evaluate the morphological characteristics of the pulmonary veins using MRA. This means that it can be synchronized with the mapping system during ablation, making it applicable to the procedure. Therefore, we believe that our method is a useful diagnostic tool, as it enables a comprehensive evaluation of the LA, including functional, morphological, and histological assessments.

## 5. Limitations

Several limitations of this study should be acknowledged. Firstly, as this is a retrospective study with a small sample size from a single institution, larger and more comprehensive prospective studies are needed to generalize the results. Secondly, recent studies have shown that additional ablation in patients with areas of LGE on MRI may not be effective in preventing recurrence [21]. Therefore, the true clinical significance of LA MRI findings remains unclear and should be further elucidated through future studies. Thirdly, due to the small number of recurrences, the number of confounders that could be included in the multivariate analysis was limited, which may have affected the adjustment for key confounding factors such as AF type, hypertension, and LA volume.

## 6. Conclusions

LA enhancement, evaluated by high-resolution LGE MRI, could be a useful imaging marker for predicting AF recurrence after catheter ablation.

## Figures and Tables

**Table 1:** Patient characteristics.

	All Patients (*n* = 100)	Recurrence (+) (*n* = 20)	Recurrence (−) (*n* = 80)	*p*-Value
Age, years	67.9 ± 9.3	67.3 ± 10.1	68.1 ± 9.3	0.751
Sex, male	78 (78.0%)	16 (80.0%)	62 (77.5%)	0.809
BMI, kg/m^2^	24.3 ± 3.6	25.4 ± 3.4	24.0 ± 3.7	0.128
Type of AF,				
Paroxysmal	50 (50.0%)	4 (20.0%)	46 (57.5%)	0.003
Persistent or long-standing persistent	50 (50.0%)	16 (80.0%)	34 (42.5%)	0.003
Time since onset of AF, months	14.5 (2.8, 46.5)	47.0 (26.3, 115.5)	11.5 (2.0, 36.0)	0.003
CHADS_2_ score	1.5 ± 1.1	1.6 ± 1.5	1.4 ± 1.0	0.557
Hypertension, n (%)	59 (59.0%)	7 (35.0%)	52 (65.0%)	0.015
Diabetes mellitus, n (%)	14 (14.0%)	5 (25.0%)	9 (11.3%)	0.113
Heart failure, n (%)	37 (37.0%)	11 (55.0%)	26 (32.5%)	0.062
Prior stroke or TIA, n (%)	5 (5.0%)	1 (5.0%)	4 (5.0%)	1.000
Coronary artery disease, n (%)	18 (18.0%)	2 (10.0%)	16 (20.0%)	0.298
Medications				
ACE-I/ARB	42 (42.0%)	8 (40.0%)	34 (42.5%)	0.839
Beta-blocker	42 (42.0%)	12 (60.0%)	30 (37.5%)	0.068
Antiarrhythmic drug before CA	19 (19.0%)	2 (10.0%)	17 (21.3%)	0.251
Hematologic test				
Hemoglobin, g/dL	14.5 ± 1.6	14.4 ± 1.6	14.5 ± 1.6	0.952
eGFR, mL/min/1.73 m^2^	62.1 ± 12.6	65.0 ± 12.6	61.4 ± 12.5	0.245
HbA1c, %	5.7 ± 0.6	5.9 ± 0.5	5.7 ± 0.5	0.121
BNP, pg/mL	127.5 ± 114.2	122.5 ± 85.2	128.8 ± 120.7	0.828
Echocardiogram				
Left atrial diameter, mm	42.0 ± 7.5	44.2 ± 5.8	41.4 ± 7.9	0.154
Left atrial volume, cc	85.5 ± 36.3	111.5 ± 46.7	79.4 ± 30.6	<0.001
Left atrial volume index, cc/m^2^	49.7 ± 20.9	62.9 ± 29.7	46.4 ± 17.0	0.001
Left ventricular ejection fraction, %	55.6 ± 13.3	51.1 ± 12.6	56.7 ± 13.3	0.092

ACE-I, angiotensin-converting enzyme inhibitor; AF, atrial fibrillation; ARB, angiotensin receptor blocker; BMI, body mass index; BNP, brain natriuretic peptide; CA, catheter ablation; eGFR, estimated glomerular filtration rate; TIA, transient ischemic attack; Values are n (%) or mean ± SD or median (interquartile range).

**Table 2:** Cardiac magnetic resonance imaging parameters.

	All Patients (*n* = 100)	Recurrence (+) (*n* = 20)	Recurrence (−) (*n* = 80)	*p*-Value
LA volume, mL	137.0 ± 46.8	167.1 ± 68.4	129.5 ± 36.9	0.001
LA wall volume, mL	71.4 ± 21.7	88.4 ± 31.0	67.2 ± 16.6	<0.001
LA wall volume with LGE ≥ 1SD, mL	9.3 ± 3.4	11.8 ± 4.4	8.6 ± 2.7	<0.001
LA wall volume with LGE ≥ 2SD, mL	1.1 ± 0.9	1.6 ± 1.1	1.0 ± 0.8	0.010
LA wall volume with LGE ≥ 3SD, mL	0.1 ± 0. 2	0.2 ± 0.3	0.1 ± 0.2	0.238
LA wall %LGE ≥ 1SD, %	12.9 ± 2.0	13.4 ± 2.0	12.8 ± 2.0	0.247
LA wall %LGE ≥ 2SD, %	1.6 ± 1.1	1.9 ± 1.1	1.5 ± 1.1	0.181
LA wall %LGE ≥ 3SD, %	0.2 ± 0.3	0.2 ± 0.3	0.2 ± 0.3	0.680

LA, left atrial, LGE, late gadolinium enhancement, SD, standard deviation; Values are n (%) or mean ± SD.

**Table 3:** Univariable Cox regression analysis for predicting recurrence after ablation.

Covariate	HR	95% CI of HR	*p*-Value
Age	0.98	0.93–1.02	0.344
Sex	1.40	0.47–4.23	0.548
Type of AF	4.93	1.65–14.79	0.004
Time since onset of AF, months	1.01	1.00–1.01	0.005
CHADS_2_ score	1.11	0.74–1.67	<0.001
LA volume by echocardiography, cc	1.02	1.01–1.03	<0.001
LA volume, mL	1.01	1.01–1.02	<0.001
LA wall volume, mL	1.03	1.02–1.04	<0.001
LA wall volume with LGE ≥ 1SD, mL	1.21	1.10–1.33	<0.001
LA wall volume with LGE ≥ 2SD, mL	1.95	1.24–3.06	0.004

AF, atrial fibrillation, CI, confidence interval, HR, hazard ratio, LA, left atrial, LGE, late gadolinium enhancement, SD, standard deviation.

**Table 4:** Multivariable Cox regression analysis for predicting recurrence.

Covariate	HR	95% CI	*p*-Value
Age	0.96	0.92–1.01	0.160
Type of AF	3.54	1.10–11.40	0.033
LA wall volume with LGE ≥1SD, per 1 mL	1.16	1.04–1.29	0.006

AF, atrial fibrillation, CI, confidence interval, HR, hazard ratio, LA, left atrial, LGE, late gadolinium enhancement, SD, standard deviation.

## Data Availability

The data presented in this study are available on request from the corresponding author due to the ethical restriction.

## Supplementary Materials

The following supporting information can be downloaded at: https://www.mdpi.com/article/10.3390/jcm13237357/s1, Table S1: Patient Characteristics between Patients with LA LGE ≥ 1SD and LA LGE < 1SD; Table S2: Additional Ablation Procedures Performed Alongside Pulmonary Vein Isolation.
