# Supplementary material for: Hyperenhancement of LA Wall by Three-Dimensional High-Resolution Late Gadolinium-Enhanced MRI and Recurrence of AF After Catheter Ablation
Source: J Clin Med. 2024 Dec 3;13(23):7357. doi: 10.3390/jcm13237357 (PMC11642366; doi:10.3390/jcm13237357)
Supplement: Supplementary file 1 [file jcm-13-07357-s001.zip › jcm-3268358-supplementary.pdf]

Supplementary Data

Hyperenhancement of LA Wall by Three-Dimensional High-Resolution Late Gadolinium-Enhanced MRI and Recurrence of AF After Catheter Ablation

Minako Kagimoto, Shingo Kato, Ryouya Takizawa, Sho Kodama, Keisuke Suzurikawa, Mai Azuma, Naoki Nakayama, Kohei Iguchi, Kazuki Fukui, Masanori Ito, Tae Iwasawa, Tabito Kino and Daisuke Utsunomiya

Supplementary Tables

Table S1. Patient Characteristics between Patients with LA LGE ≥ 1SD and LA LGE < 1SD. 1

Table S2. Additional Ablation Procedures Performed Alongside Pulmonary Vein Isolation. 3

Table S1. Patient Characteristics between Patients with LA LGE ≥ 1SD and LA LGE < 1SD.

	**All patients (*n* = 100)**	**LA LGE ≥ 1SD (*n* = 22)**	**LA LGE < 1SD (*n* = 78)**	***p*-Value**
Age, years	67.9 ± 9.3	66.5 ± 10.5	68.3 ± 9 .1	0.462
Sex, male	78 (78.0%)	18 (81.8%)	60 (76.9%)	0.625
BMI, kg/m^2	24.3 ± 3.6	25.8 ± 3.5	23.9 ± 3.6	0.035
Type of AF,				
Paroxysmal	50 (50.0%)	4 (18.2%)	46 (59.0%)	< 0.001
Persistent or long-standing persistent	50 (50.0%)	18 (81.8%)	32 (41.0%)	< 0.001
Time since onset of AF, months	14.5 (2.8, 46.5)	32.5 (12.5, 54.8)	12.0 (2.0, 38.5)	0.022
$CHADS_2$ score	1.5 ± 1.1	1.5 ± 0.9	1.5 ± 1.1	0.818
Hypertension, n (%)	59 (59.0%)	11 (50.0%)	48 (61.5%)	0.331
Diabetes mellitus, n (%)	14 (14.0%)	2 (9.1%)	12 (15.4%)	0.452
Heart failure, n (%)	37 (37.0%)	10 (45.5%)	27 (34.6%)	0.352
Prior stroke or TIA, n (%)	5 (5.0%)	1 (4.5%)	4 (5.1%)	0.912
Coronary artery disease, n (%)	18 (18.0%)	2 (9.1%)	16 (20.5%)	0.218
AF recurrence, n (%)	20 (20.0%)	11 (50.0%)	9 (11.5%)	<0.001
Medications				
ACE-I / ARB	42 (42.0%)	10 (45.5%)	32 (41.0%)	0.710
Beta-blocker	42 (42.0%)	12 (54.5%)	30 (38.5%)	0.177
Antiarrhythmic drug before CA	19 (19.0%)	4 (18.2%)	15 (19.2%)	0.912
Hematologic test				
Hemoglobin, g/dL	14.5 ± 1.6	14.8 ± 1.6	14.4 ± 1.5	0.300
eGFR, mL/min/1.73m^2	62.1 ± 12.6	62.4 ± 14.7	62.0 ± 12.0	0.884
HbA1c, %	5.7 ± 0.6	5.6 ± 0.4	5.7 ± 0.6	0.344
BNP, pg/ml	127.5 ± 114.2	141.5 ± 100.8	123.6 ± 118.0	0.518
Echocardiogram				
LA diameter, mm	42.0 ± 7.5	45.2 ± 6.5	41.1 ± 7.6	0.022
LA volume, cc	85.5 ± 36.3	114.0 ± 42.5	77.8 ± 30.5	< 0.001
LA volume index, cc/m^2	49.7 ± 20.9	62.6 ± 23.3	46.1± 19.0	< 0.001
LV ejection fraction, %	55.6 ± 13.3	56.0 ± 9.7	55.5 ± 14.2	0.869

ACE-I, angiotensin-converting enzyme inhibitor; AF, atrial fibrillation; ARB, angiotensin receptor blocker; BMI, body mass index; BNP, brain natriuretic peptide; CA, catheter ablation; eGFR, estimated glomerular filtration rate; LA, left atrial, LGE, late gadolinium enhancement; TIA, transient ischemic attack; Values are n (%) or mean ± SD or median (interquartile range). *p*-Value represents significance of difference between patients with LA LGE ≥ 1SD and LA LGE < 1SD.

Table S2. Additional Ablation Procedures Performed Alongside Pulmonary Vein Isolation.

	**All patients (n = 100)**	**Recurrence (+) (n = 20)**	**Recurrence (–) (n = 80)**	***p*-Value**
Additional ablation procedures	22 (22.0%)	9 (45.0%)	13 (16.3%)	0.006
+ SVCI	1 (1.0%)	0 (0.0%)	1 (1.3%)	0.615
+ BOXI	20 (20%)	8 (40.0%)	12 (15.0%)	0.012
+ SVCI + BOXI	1 (1.0%)	1 (5.0%)	0 (0.0%)	0.044

BOXI, box isolation; SVCI, superior vena cava isolation; Values are n (%). *p*-Value represents significance of difference between patients with recurrence (+) and recurrence (–).
